# A Versatile Luminescent Ga-Organic Framework with Multi-Emission Centers as a Blue LED and Fluorescent Probe for Low-Temperature Detection and Selective Fe^3+^ Sensing

**DOI:** 10.3390/nano12224009

**Published:** 2022-11-15

**Authors:** Weiwei Shi, Lei Liang, Jinping Zhang, Haihan Ye, Xincheng Hu, Jianwei Zhang, Wei Wei

**Affiliations:** 1 School of Petrochemical Engineering, Liaoning Petrochemical University, Fushun 113001, China; 2 Henan Engineering Center of New Energy Battery Materials, School of Chemistry and Chemical Engineering, Shangqiu Normal University, Shangqiu 476000, China

**Keywords:** Ga-organic framework, multi-emission centers, blue LED, low-temperature sensing, Fe^3+^ sensing

## Abstract

The development and utilization of 3p-block based MOFs as fluorescent materials has attracted significant attention in recent years. Herein, we have successfully constructed a versatile luminescent Ga-MOF (SNNU-63) with a 3d^10^ configuration and a large ligand twist configuration. Interestingly, the as-synthesized Ga-MOF exhibits excellent luminescence property and a good material for blue light-emitting diode (LED). At 80 K, this Ga-MOF shows multi-emission centers at 381, 462, and 494 nm. As a ratiometric thermometer, this Ga-MOF exhibits an excellent temperature sensing property with high relative sensitivity (*S*_m_ = 2.60 % K^−1^ at 110 K). The fluorescence intensity ratio *I*_381_/*I*_494_ shows a very good fit for the Boltzmann results (80–240 K). Moreover, the luminescent Ga-MOF exhibits an excellent selective detection of Fe^3+^ over other metal ions in aqueous an medium, and the limit of detection (LOD) towards Fe^3+^ ions is calculated to be 1.227 × 10^−4^ M. This work presents a versatile luminescent Ga-MOF material as a blue LED and fluorescent probe for low-temperature and selective Fe^3+^ sensing.

## 1. Introduction

Luminescent metal–organic frameworks (LMOFs) with their unique photoluminescence properties have attracted much research interest because of their potential applications in light emission, biological imaging, and fluorescence sensing, etc. [1,2,3,4,5,6,7,8,9,10]. It is generally believed that a ligand-based emission strategy is the most direct way to construct high-performance fluorescent MOF materials without considering rare-earth based MOFs. Recently, we and other groups have found that the fluorescence performance can be improved when the delocalized conjugate system is broken, which is mainly attributed to the strong coordination bonds and the torsion between aromatic rings [11,12,13,14]. Therefore, it is still significant to study the construction of the fluorescent MOFs materials by an aromatic torsion strategy.

In practical applications, the stable MOF materials should be considered when the water or acid/base research system appears. To our knowledge, the strong coordination bonds, such as the bonding between the high valent metal ion (hard Lewis acid) and the carboxylate ligand (hard base), can resist the attack of water and acidic or basic reactants [15,16,17,18,19,20]. On the other hand, the fluorescent MOFs with unique multi-emission centers opens up the new approach to develop and explore new LMOFs materials for ratiometric fluorescent sensing [21,22,23,24,25,26].

Through the above discussion, a highly stable Ga-MOF as reported in our previous work, namely, [Ga_3_(*μ*_3_-O)(PTC)_2_(CH_3_COO)(H_2_O)] (SNNU-63), has potential as a versatile fluorescent material because it has unique structural characteristics, such as the 3d^10^ configuration and a large ligand twist configuration (Figure S1) [27]. As predicted, this fluorescent material not only exhibits excellent fluorescence performance but also is widely used in many fields and applications, such as in blue LEDs, ratiometric thermometers, and selective Fe^3+^ sensing.

## 2. Experimental Section

Synthesis of SNNU-63. A mixture of Ga(NO_3_)_3_·xH_2_O (150 mg, 0.59 mmol) and the organic ligand H_3_PTC (100 mg, 0.35 mmol, H_3_PTC = biphenyl-3,4′,5-tricarboxylic acid), DMF (4 mL, DMF = N,N-dimethylformamide) and acetic acid (0.5 mL) was sealed in the vial (20 mL), then heated at 130 °C for 72 h [16]. The yellow colorless rodlike crystals were obtained by filtering and washing several times with fresh DMF and ethanol, then dried in air (80.0% yield based on the ligand). 

## 3. Results and Discussion

**Figure 1:**
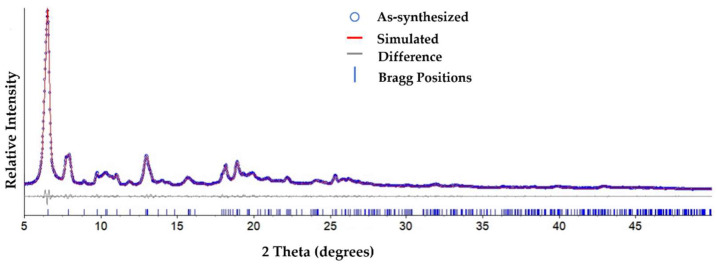
The Rietveld refinement XRD pattern of the as-synthesized SNNU-63.

In the process of preparing Ga-MOF (SNNU-63), we expanded the mass production. Compared with our previous report, we input about 5 times the amount of raw materials and 2 times the amount of solvent DMF. To better determine the phase purity of as-synthesized SNNU-63, The as-synthesized samples were investigated on XRD Rietveld refinement by using the software Topas 5 (Figure 1) [28,29]. The results of Rietveld refinement for as-synthesized SNNU-63 show that all the refined crystallographic parameters well match the structural model obtained by single-crystal analysis, and good fits were obtained with *R*_p_ = 2.63, *R*_wp_ = 3.27 and GOF = 1.17.

Compared with the FT-IR spectrum of the ligand, the disappearance of 1693 cm^−1^ and the appearance of 1621 cm^−1^ for SNNU-63 indicate that carboxylate groups of the ligand are fully protonated, that is, the carboxyl group are completely coordinated with the metal, which is consistent with the result of single crystal structure (Figure S2). 

### 3.1. Photoluminescence Study

**Figure 2:**
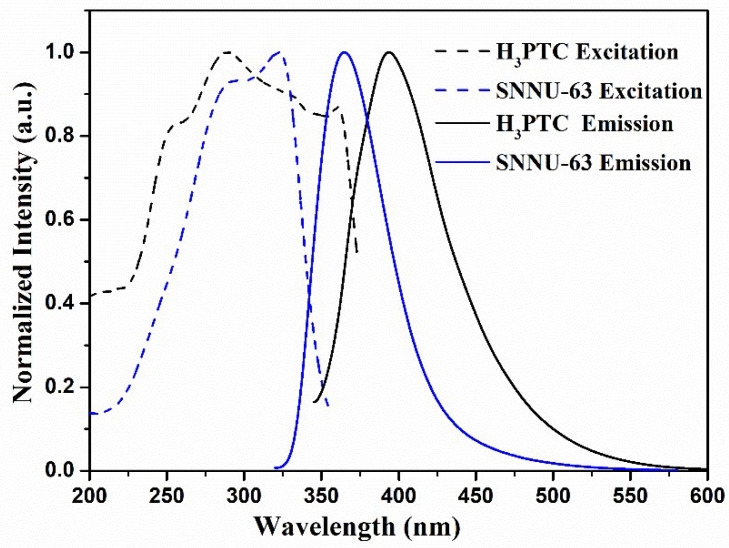
Solid-state excitation and emission spectra of SNNU-63 and the free H_3_PTC ligand.

Considering that SNNU-63 has a unique structure, like the 3d^10^ configuration and a large ligand twist configuration, we measured the solid-state photoluminescence properties of the free ligand (H_3_PTC) and SNNU-63. Solid-state excitation and emission spectra at room temperature are shown in Figure 2. The free ligand shows the maximum emission peak at 393 nm under the 325 nm excitation, while SNNU-63 exhibits the maximum emission peak at 364 nm under the 300 nm excitation. Clearly, the maximum emission of SNNU-63 exhibits a blue-shift of 29 nm compared to that of the free ligand, which may arise from the different ligand conformations within SNNU-63 and H_3_PTC. To our knowledge, the ligand has a larger twist in the MOF skeleton compared to the free ligand, which can cause the MOF to have higher energy UV absorption than the free ligand. As predicted, the solid-state UV absorption maxima of the free ligand and SNNU-63 are at 325 and 300 nm, respectively (Figure S3). A blue shift (25 nm) of UV-vis absorption peak is consistent with the solid-state fluorescence’s result. Therefore, we propose that the blue-shift of the electronic absorption and emission spectra might be caused by different ligand twisted conformations within SNNU-63 and H_3_PTC [11,13]. Moreover, the CIE chromaticity coordinate of SNNU-63 is located in the blue region (x = 0.1584, y = 0.0566) (Figure S4), and the as-synthesized SNNU-63 exhibits the blue color under 254 nm UV light (Figure S5). Both the CIE coordinate and luminescent photo indicate SNNU-63 as a promising material for blue light-emitting diodes (LEDs).

### 3.2. Temperature-Dependent Emission Properties

**Figure 3:**
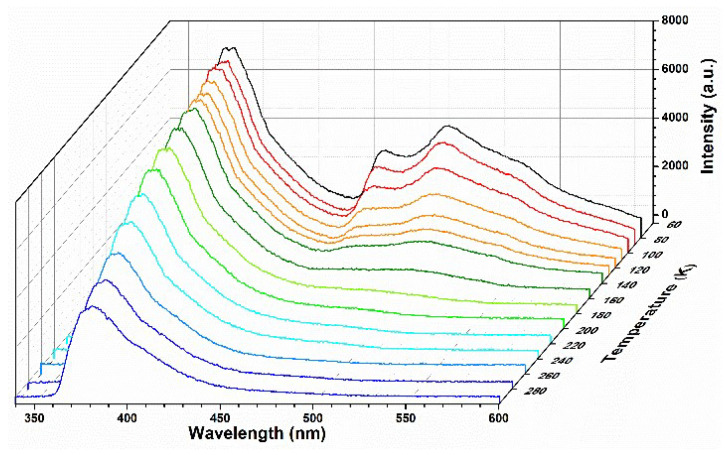
Temperature-dependent emission spectra of SNNU-63 in the range of 80–300 K.

Considering SNNU-63 with unique the 3d^10^ configuration and the large ligand twisted conformation, we also investigated its temperature-dependent emission spectra in the range of 80–300 K (Figure 3). Obviously, when the temperature drops to 80 K, the MOF exhibits unique multi-emission centers, i.e., 381, 462, and 494 nm. This MOF at low temperature shows that the multi-emission centers are mainly derived from the limited ligand twisted conformations, and the reduced non-radiative attenuation for the benzene rings. When the temperature rises, the fluorescence intensities (*I*) of 462 and 494 nm gradually disappear, and only 381 nm is the main characteristic peak at 300 K. 

**Figure 4:**
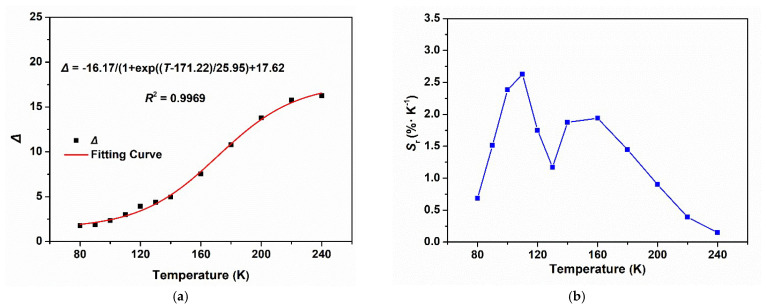
(**a**) Temperature-dependent emission intensity ratio Δ(*I*_493_/*I*_410_) and the fitted curve of SNNU-63 in the range of 80–240 K; (**b**) temperature-dependent generalized relative sensitivity (S_r_) of SNNU-63.

The unique multi-emission centers of SNNU-63 at low temperatures have encouraged us to evaluate its application as a ratiometric thermometer in low-temperature detection. Here, we investigated the emission intensities at 381 and 494 nm to establish its potential as a ratiometric thermometer when excited 300 nm. The fluorescence intensity ratio *I*_381_/*I*_494_ as the self-calibrated thermometric parameter (Δ) with temperature was evaluated. As shown in Figure 4a, the temperature dependence of the fluorescence intensity ratio *I*_381_/*I*_494_ can be described by the Boltzmann equation (Δ = (A_1_ − A_2_)/(1 + exp((*T* − *T*_0_)/d*T*)) + A_2_) (fitting parameters: A_1_ = 1.45, A_2_ = 17.62, *T*_0_ = 171.22, d*T* = 25.95), and a very good fit between Δ and temperature (80–240 K) (correlation coefficient *R*^2^ = 0.9969) can be obtained. These results demonstrate that SNNU-63 is a desirable candidate for highly sensitive temperature sensing. 

To evaluate its practical application as a temperature sensor, we further investigated the relative sensitivity (Sr, Sr = (∂Δ/∂T)/Δ) in the range of 80–240 K. The calculated maximum relative sensitivity *S*_m_ = 2.60% K^−1^ is observed at 110 K (Figure 4b), which is comparable to that of the other reported MOFs with bimetallic Ln-MOF thermometers such as Tb_0_._80_Eu_0_._20_BPDA (1.19) [30], Nd_0_._577_Yb_0_._423_BDC-F_4_ (1.2) [31], and Eu_0_._37_Tb_0_._63_-BTC-a (0.68) [32]. Hence, this Ga-MOF is a good platform for temperature sensing in a wide temperature range.

### 3.3. Metal Ions Sensing Measurements

**Figure 5:**
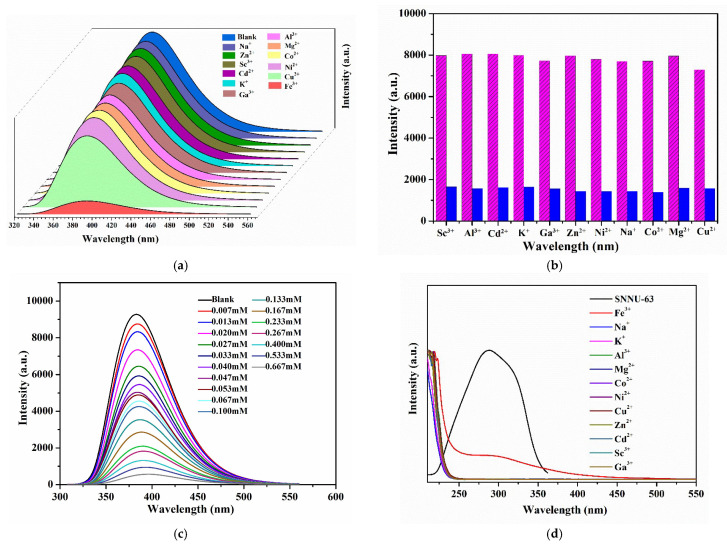
(**a**) Fluorescent spectra of SNNU-63 in the presence of 0.4 mM of different metal ions under an excitation of 288 nm. (**b**) The anti-interference performance of the coexistence of other metal ions and Fe^3+^ ions for SNNU-63. (**c**) Luminescence spectra of SNNU-63 toward Fe^3+^ ions with different concentrations of Fe^3+^ ranging from 0 to 0.667 mM. (**d**) Luminescence excitation spectrum of SNNU-63 suspensions and UV-vis absorption spectra of twelve different metal ion solutions.

Owning to the excellent water stability and fluorescence properties of SNNU-63, we investigated the fluorescence response experiments of this MOF towards metal ions. The excitation and emission spectra of this MOF aqueous suspension are shown in Figure S6, i.e., the maximum emission intensity at 382 nm upon excitation at 288 nm. The metal ion sensing, based on SNNU-63, was performed by adding 2 mL aqueous solutions of metal salts (0.6 mM) (M^n+^ = Sc^3+^, Al^3+^, Cd^2+^, K^+^, Ga^3+^, Zn^2+^, Ni^2+^, Na^+^, Co^2+^, Mg^2+^, Cu^2+^, Fe^3+^) with a 1 mL suspension of SNNU-63 (1 mg/mL). Clearly, the fluorescence emission intensity at 383 nm of SNNU-63 toward Fe^3+^ ion exhibits the highest quenching efficiency, while others metal ions show negligible effect on the fluorescence intensities (Figure 5a and Figure S7). It is noteworthy that the fluorescence quenching rate of this MOF towards Fe^3+^ ion is as high as 87% (Figure S8). The result demonstrates that SNNU-63 exhibits the high selectivity to Fe^3+^ ions in aqueous media via fluorescence quenching.

Considering that other metal ions coexist with Fe^3+^ ion in practical applications, the fluorescent emission intensities of SNNU-63 with different mixed ions before and after Fe^3+^ ion incorporation (0.3 mM) were recorded and compared (Figure 5b). Even in the presence of other metal ions, the fluorescence quenching efficiency of SNNU-63 towards Fe^3+^ ion was obvious. Hence, SNNU-63 exhibits a high selectivity for detecting Fe^3+^ ion in aqueous media.

To further investigate the detection limit and sensitivity for SNNU-63 toward Fe^3+^ ions, concentration dependence experiments were performed by adding different concentrations of Fe^3+^ ions, ranging from 0 to 0.667 mM (Figure 5c). It was very obvious that the luminescence intensity (*I*_382nm_) of SNNU-63 gradually decreased with the increase of the Fe^3+^ concentration. At a low concentration (0–0.04 mM) (Figure S9), the fluorescence quenching constant (*K*_sv_) was used in the Stern–Volmer (SV) equation: (*I*_0_/*I*) = 1 + *K*_sv_[M], where *I*_0_ and *I* are the luminescence intensity of SNNU-63 suspensions before and after adding different concentrations of Fe^3+^ ions, *K*_sv_ is the SV constant, and [M] is the molar concentration of Fe^3+^ ions. The *K*_sv_ value was evaluated to be 2.259 × 10^4^ M^−1^, which is comparable to that of the other stable MOFs toward Fe^3+^ sensing, such as BUT-15 (1.66 × 10^4^ M^−1^) [18] and Tb–HIAAC (2.5 × 10^4^ M^−1^) [33]. Based on the 3*S*_b_/*K*_sv_ equation, the limit of detection (LOD) of SNNU-63 towards Fe^3+^ ion was evaluated to be 1.227 × 10^−4^ M. Overall, low LOD and high *K*_sv_ values enable SNNU-63 to detect Fe^3+^ with high sensitivity and accuracy. As a fluorescent probe, the reversible and recyclable experiments of SNNU-63 toward Fe^3+^ ion should be investigated. The result of four cycles demonstrates that SNNU-63 exhibits an excellent candidate for detecting Fe^3+^ with good recyclability and stability (Figure S10).

The rapid fluorescence quenching response and high selectivity of Fe^3+^ ion sensing for SNNU-63 prompted us to explore its fluorescence sensing mechanism. As shown in Figure 1, we determined that MOF materials can stably exist in the Fe^3+^ sensing system, which was proved by PXRD (Figure S11). Generally, the photoinduced electron transfer (PET) process can occur when the excited electrons of MOF are transferred to the LUMO of Fe^3+^ ion upon excitation [18]. On the other hand, a broad excitation of SNNU-63 showed the greatest degree of overlapping the absorption band of Fe^3+^ ions. However, no clear overlapping was observed between the excitation of SNNU-63 and the absorption spectra of other metal ions (Figure 5d). This result shows that the SNNU-63 towards Fe^3+^ sensing conforms to the fluorescence resonance energy transfer (FRET) process. Hence, the mechanism of stable SNNU-63 towards Fe^3+^ sensing is mainly attributed to both the PET and FRET processes.

## 4. Conclusions

In summary, a versatile luminescent Ga-MOF (SNNU-63) constructed with a 3d^10^ configuration and a large ligand twist configuration has been synthesized and studied. Due to the synergism between the metal center and ligand within the framework, this Ga-MOF should have excellent fluorescence performance and can be used for light emission and fluorescence sensing. The as-synthesized Ga-MOF exhibits a blue-shift of 29 nm compared to that of the free ligand. The CIE coordinate and luminescent photo demonstrate that this Ga-MOF is a promising material for blue light-emitting diodes. More importantly, this Ga-MOF exhibits multi-emission centers at 381, 462, and 494 nm at 80 K. The temperature dependence of fluorescence intensity ratio *I*_381_/*I*_494_ and the relative sensitivity analyses demonstrate that this Ga-MOF exhibits an excellent temperature sensing capability (80–240 K). In addition, the stable SNNU-63 exhibits an excellent selective detection of Fe^3+^ over other metal ions in aqueous medium. Overall, our work provides an effective method to develop and utilize luminescent Ga-MOFs materials.

## Supplementary Materials

The following supporting information can be downloaded at: https://www.mdpi.com/article/10.3390/nano12224009/s1, Figure S1: Structure of the organic ligand (H_3_PTC). Figure S2: FT-IR spectra of the organic ligand and SNNU-63. Figure S3: The solid-state UV-Vis absorption spectra of SNNU-63 and the free H_3_PTC ligand at room temperature. Figure S4: The corresponding CIE 1931 chromaticity diagram of SNNU-63 at room temperature. Figure S5: Comparison of photo for SNNU-63 under the natural light (a) and UV 254 nm light (b). Figure S6: Excitation and emission spectra of SNNU-63 MOF aqueous suspension. Figure S7: Luminescence intensity (*I*_382 nm_) of SNNU-63 to different metal ions in aqueous (0.4 mM) under excitation of 288 nm. Figure S8: Fluorescence quenching of SNNU-63 dispersed in aqueous solutions of twelve different metal cations (0.4 mM). Figure S9: Linearity relationship of luminescent intensity of SNNU-63 and Fe^3+^ ion at low concentrations. Figure S10: The reversibility and recyclability of SNNU-63 based on the maximum emission intensity at 382 nm in water (1 mL 2 mg/mL) in the presence of 2 mL of a 0.6 mM aqueous solution of Fe^3+^ ions. Figure S11: Comparison of PXRD patterns of the as-synthesized MOF and the MOF after Fe^3+^ sensing.
