# Supplementary material for: A Versatile Luminescent Ga-Organic Framework with Multi-Emission Centers as a Blue LED and Fluorescent Probe for Low-Temperature Detection and Selective Fe3+ Sensing
Source: Nanomaterials (Basel). 2022 Nov 15;12(22):4009. doi: 10.3390/nano12224009 (PMC9696890; doi:10.3390/nano12224009)
Supplement: Supplementary file 1 [file nanomaterials-12-04009-s001.zip › nanomaterials-2042249-supplementary.pdf]

Supporting Information

A Versatile Luminescent Ga-Organic Framework with Multi-Emission Centers as a Blue LED and Fluorescent Probe for Low-Temperature Detection and Selective Fe^{3+} Sensing

Weiwei Shi [1], Lei Liang [1], Jinping Zhang [2], Haihan Ye [2], Xincheng Hu [2], Jianwei Zhang [2,*] and Wei Wei [2]

[1] School of Petrochemical Engineering, Liaoning Petrochemical University, Fushun 113001, China
[2] Henan Engineering Center of New Energy Battery Materials, School of Chemistry and Chemical Engineering, Shangqiu Normal University, Shangqiu 476000, China
* Correspondence: jwzhang85@163.com

Citation: Shi, W.; Liang, L.; Zhang, J.; Ye, H.; Hu, X.; Zhang, J.; Wei, W. A Versatile Luminescent Ga-Organic Framework with Multi-Emission Centers as a Blue LED and Fluorescent Probe for Low-Temperature Detection and Selective Fe^{3+} Sensing. *Nanomaterials* **2022**, *12*, 4009. https://doi.org/10.3390/nano12224009

Academic Editor: Vincenzo Vaiano

Received: 3 November 2022
Accepted: 11 November 2022
Published: 15 November 2022

Publisher's Note: MDPI stays neutral with regard to jurisdictional claims in published maps and institutional affiliations.

Copyright: © 2022 by the authors. Submitted for possible open access publication under the terms and conditions of the Creative Commons Attribution (CC BY) license (https://creativecommons.org/licenses/by/4.0/).

1. Materials and Methods.

Reagents: All reagents and solvents were purchased from commercial sources without further purification. The organic ligand biphenyl-3,4′,5-tricarboxylic acid (H_3PTC, 97%) were purchased from CHEMSOON Co., Ltd (Shanghai, China). $Ga(NO_3)_3{\cdot}xH_2O$ (99.9%), $Sc(NO_3)_3{\cdot}xH_2O$ (99.99%), DMF (N,N-dimethylformamide, 99.5%) and HAC (acetic acid, 99.5%) were purchased from Aladdin Industrial Inc (Shanghai, China). $NaNO_3$ (AR), KNO_3 (AR), $Cd(NO_3)_2{\cdot}4H_2O$ (AR), $Co(NO_3)_2{\cdot}6H_2O$ (AR), $Zn(NO_3)_2{\cdot}6H_2O$ (AR), $Mg(NO_3)_2{\cdot}6H_2O$ (AR), $Al(NO_3)_3{\cdot}9H_2O$ (AR), $Fe(NO_3)_3{\cdot}9H_2O$ (AR), $Ni(NO_3)_2{\cdot}6H_2O$ (AR), EtOH (ethanol, AR), $Cu(NO_3)_2{\cdot}3H_2O$ (AR), $BaSO_4$ (AR) and KBr (AR) were purchased from Sinopharm Chemical Reagent Co., Ltd (Shanghai, China).

Characterization: Powder X-ray diffraction (PXRD) pattern was collected by a Rigaku MiniFlex600 diffractometer with Cu Kα (λ =1.54056 Å, 40 kV, 15 mA) at room temperature by 0.02^{o} steps. FT-IR spectra were recorded on a Bruker Tensor 27 spectrophotometer using KBr pellets in the range of 4000–400 cm^{-1}. The UV-vis absorption spectra measured on a Hitachi U-3900 UV/vis spectrophotometer (PMT voltage: Auto, Slit width: 2 nm, Lamp change mode: Auto, Scan speed: 1200 nm/min) using $BaSO_4$ pellets in the range of 700–200 nm at room temperature. The temperature-dependent luminescent measurements were recorded a Horiba FluoroMax+ spectrofluorometer (EM1: IHR 320 (Mono1), Side entrance slit: 1.60 nm bandpass, Side exit slit: 1.60 nm bandpass, Grating: density 150 (blaze: 500), Start: 589.33nm, End: 589.33nm; EX1: 180 DF (Mono2), Park: 300.00nm, Side entrance slit: 1.60 nm bandpass, Side exit slit: 1.60 nm bandpass, First intermediate slit: 1.60 nm bandpass, Grating: density 1200 (blaze: 330)). The solid-state photoluminescence properties were recorded on Hitachi F-7100 fluorescence spectrophotometer (PMT voltage: 400 V, EX slit: 5.0 nm, EM slit: 5.0 nm, EX WL: 325.0 nm, Scan speed:1200 nm/min for H_3PTC; PMT voltage: 400 V, EX slit: 2.5 nm, EM slit: 2.5 nm, EX WL: 300.0 nm, Scan speed:1200 nm/min for SNNU-63) at room temperature. Metal ions sensing were recorded on Hitachi F-7100 fluorescence spectrophotometer (PMT voltage: 700 V, EX slit: 2.5 nm, EM slit: 2.5 nm, EX WL: 288.0 nm, Scan speed:1200 nm/min) at room temperature.

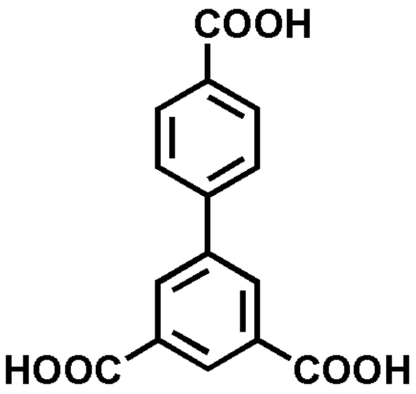

Figure S1. Structure of the organic ligand (H_3PTC).

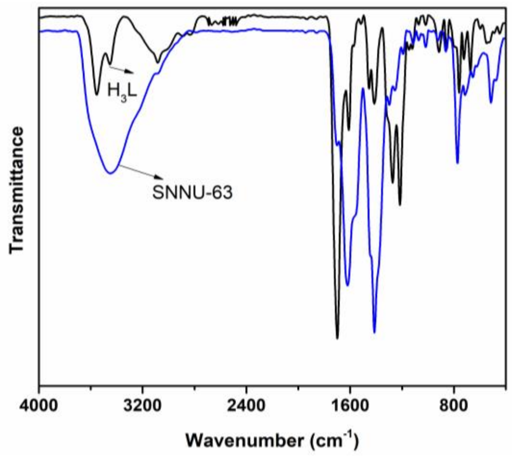

Figure S2. FT-IR spectra of the organic ligand and SNNU-63.

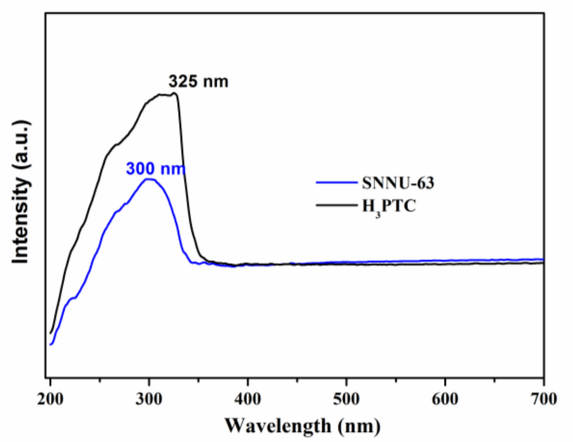

Figure S3. The solid-state UV-Vis absorption spectra of SNNU-63 and the free H_3PTC ligand at room temperature.

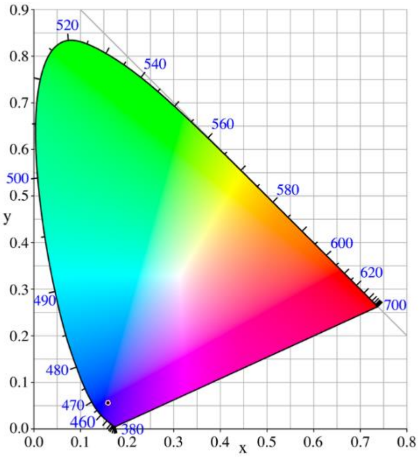

Figure S4. The corresponding CIE 1931 chromaticity diagram of SNNU-63 at room temperature.

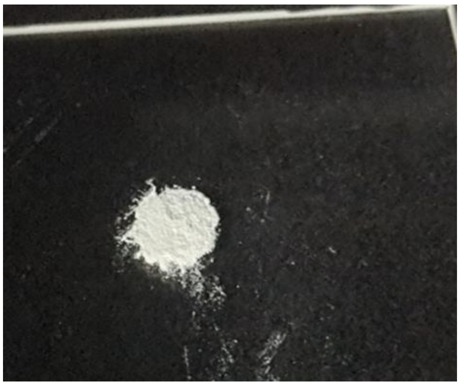

(a)

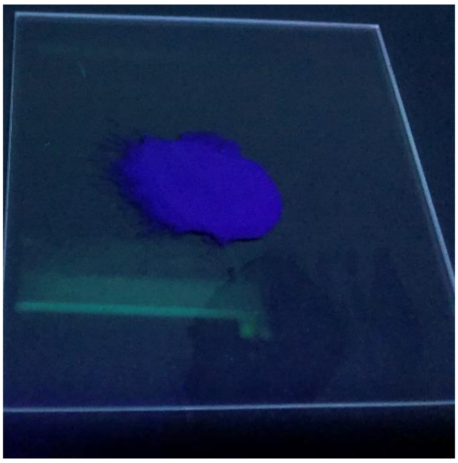

(b)

Figure S5. Comparison of photo for SNNU-63 under the natural light (a) and UV 254 nm light (b).

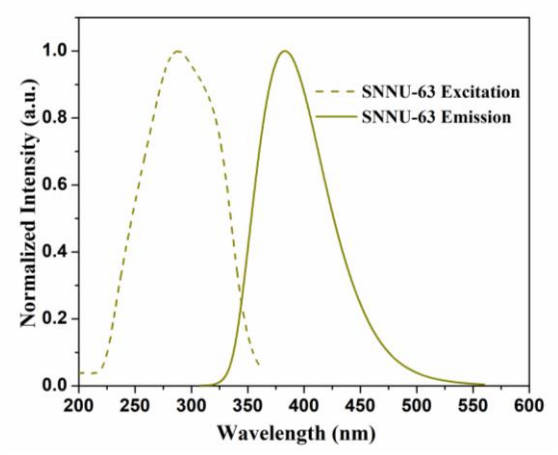

Figure S6. Excitation and emission spectra of SNNU-63 MOF aqueous suspension.

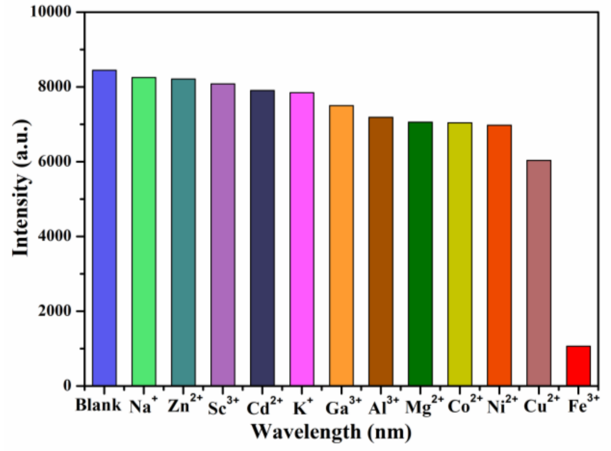

Figure S7. Luminescence intensity ($I_{382\ nm}$) of SNNU-63 to different metal ions in aqueous (0.4 mM) under excitation of 288 nm.

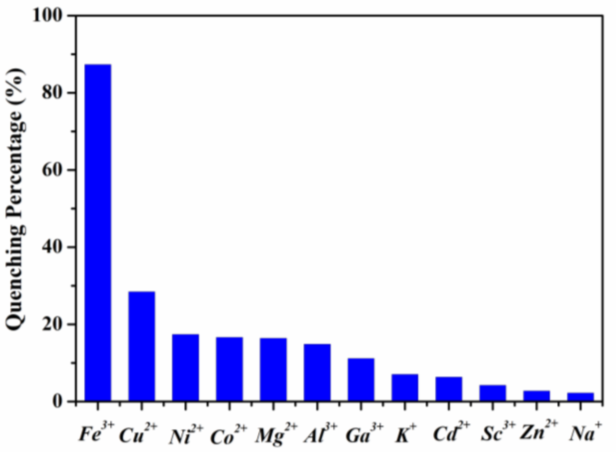

Figure S8. Fluorescence quenching of SNNU-63 dispersed in aqueous solutions of twelve different metal cations (0.4 mM).

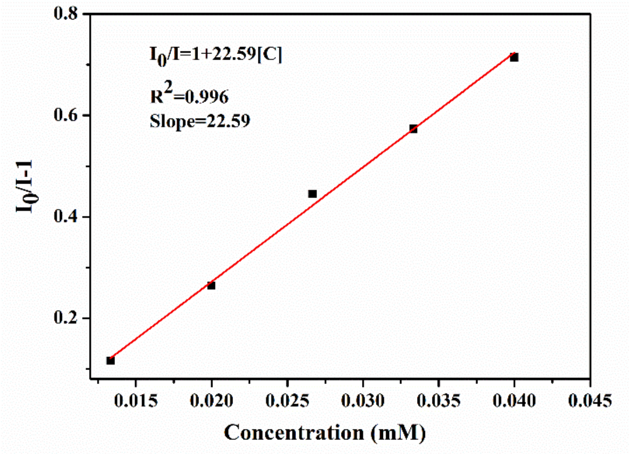

Figure S9. Linearity relationship of luminescent intensity of SNNU-63 and Fe^{3+} ion at low concentrations.

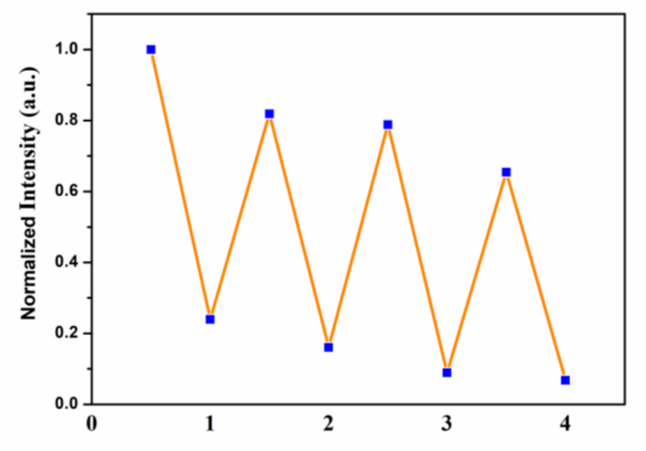

Figure S10. The reversibility and recyclability of SNNU-63 based on the maximum emission intensity at 382 nm in water (1 mL 2 mg/mL) in the presence of 2 mL 0.6 mM aqueous solution of Fe^{3+} ions.

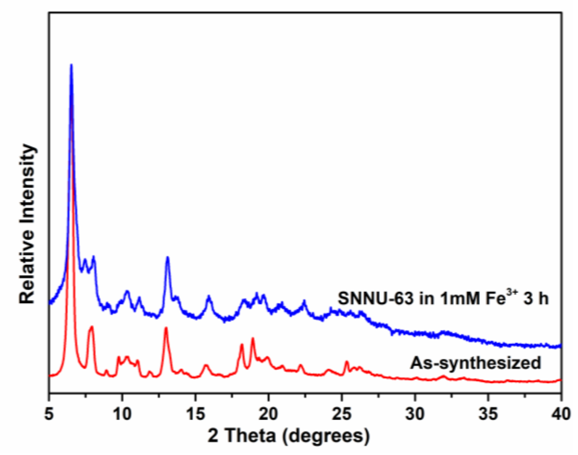

Figure S11. Comparison of PXRD patterns of as-synthesized MOF and the

MOF after Fe^{3+} sensing.
